# Prevalence, risk factors, and management practices of primary dysmenorrhea among young females

**DOI:** 10.1186/s12905-021-01532-w

**Published:** 2021-11-08

**Authors:** Samar Karout, Lama Soubra, Deema Rahme, Lina Karout, Hani M J Khojah, Rania Itani

**Affiliations:** 1 Pharmacy Practice Department, Faculty of Pharmacy, Beirut Arab University, Riad El Solh, P.O. Box 11-5020, Beirut, 1107 2809 Lebanon; 2 Pharmacology and Therapeutics Department, Faculty of Pharmacy, Beirut Arab University, Riad El Solh, P.O. Box 11-5020, Beirut, 1107 2809 Lebanon; 3 Department of Radiology, American University of Beirut Medical Center, Riad El-Solh, P.O. Box 11-0236, Beirut, 1107 2020 Lebanon; 4 Department of Clinical and Hospital Pharmacy, College of Pharmacy, Taibah University, Madinah, Kingdom of Saudi Arabia

**Keywords:** Primary dysmenorrhea, Menstrual pain, Prevalence, Risk factors, Management-seeking practices, Non-steroidal anti-inflammatory drugs, Lebanon

## Abstract

**Background:**

Primary dysmenorrhea (PD) is one of the most common gynecological conditions among young females, which has a significant negative impact on health-related quality of life and productivity. Despite its high prevalence, the evidence is limited regarding the management-seeking practices and its perceived effectiveness among females with PD.

**Methods:**

This is a cross-sectional study conducted among 550 female students in six universities across Lebanon. The prevalence of PD, associated risk factors, and management-seeking practices were assessed using a self-administered questionnaire.

**Results:**

The prevalence of PD was 80.9%. Most of the females with PD described their menstrual pain as moderate (56%) to severe (34.6%), which significantly affected their daily activities and studying ability (*P* < 0.001). The major risk factors associated with PD included heavy menstrual flow (adjusted odds ratio [AOR] = 10.28), family history of PD (AOR = 2.52), history of weight loss attempt (AOR = 2.05), and medical specialization (AOR = 1.663). Only 36.9% of females with PD sought formal medical advice. Most dysmenorrheic females (76.4%) received medications for the management of PD, and remarkably none of them took hormonal contraceptives. Drugs commonly used for PD were mefenamic acid (26.2%), ibuprofen (25%), and paracetamol (11.5%), which were administered when the pain started (58.2%). All medications were significantly effective in reducing the pain score (*P* = 0.001), and most NSAIDs were more potent than paracetamol in managing PD (*P* = 0.001). However, no significant difference in adverse effects among medications was revealed. Moreover, no superiority of any individual NSAID for pain relief was established. Nevertheless, mefenamic acid was associated with the lowest risk of abdominal pain (OR: 0.03, *P* = 0.005) and the highest risk of flank pain (OR = 12, *P* = 0.02).

**Conclusions:**

Suboptimal management of PD is practiced among university students in Lebanon. Therefore, health care providers should educate dysmenorrheic females to optimize the self-management support of PD. Furthermore, future research is required to investigate females’ misconceptions about hormonal contraceptives in the management of PD, aiming to raise awareness and correct misconceptions.

**Supplementary Information:**

The online version contains supplementary material available at 10.1186/s12905-021-01532-w.

## Introduction

Dysmenorrhea is one of the most common gynecological conditions that most of child-bearing females suffer from during menstruation [1]. Although it is a common condition, it is usually under-diagnosed, and most women do not seek medical attention [2, 3]. Dysmenorrhea is defined as a cyclic lower abdominal or pelvic pain that may be radiating to the lower back, legs and inner thighs [4]. It is classified into primary dysmenorrhea (PD) and secondary dysmenorrhea (SD). PD refers to menstrual pain without any obvious pelvic pathology, when the ovulatory cycle is established, which typically begins within the first two years after menarche [1, 5]. On the other hand, secondary dysmenorrhea is the menstrual pain associated with underlying pelvic pathology such as endometriosis, chronic pelvic inflammatory disease, adenomyosis, endometrial polyps, and complications of intrauterine contraceptive device [1, 4].

The worldwide prevalence of PD ranges from 45 to 95% of women of reproductive age, where 2–29% experience severe pain [1, 4]. The national prevalence of PD in Lebanon ranges between 38.1 and 89.6%, with a sample of different age groups and occupation [6–8]. This variation is explained by different methodologies used to assess PD, selected population, age group, ethnicity, and pain perception difference among communities.

PD has a significant negative impact on health-related quality of life, daily activities, work productivity and academic performance [1, 9–11]. About 42% of young women reported limitation in daily activities, and 17% reported missing 1–2 days of work or school [9]. Despite its high prevalence and impact on daily activities, PD is often inadequately treated and even disregarded since many young females suffer silently without seeking medical advice. Females find PD embarrassing and taboo, and they perceive it as an inevitable response to menstruation where its pain should be tolerated [12, 13]. Several factors have been proven to increase the risk of dysmenorrhea such as early menarche, nulliparity, irregular menstrual cycle, long menstrual duration, heavy menstrual flow, family history of dysmenorrhea, and smoking [9]. Whereas, factors that may reduce the risk and severity of dysmenorrhea include normal child delivery and physical exercise [14].

The PD pain usually starts 1–2 days before the onset of menses, or just after the menstrual flow [5], and pain typically lasts 8–72 h [15]. In addition to the lower abdominal/pelvic pain, dysmenorrhea is usually associated with common symptoms that can be categorized into two main dimensions—physical and psychological. The commonly experienced physical symptoms are systemic, gastrointestinal, and eliminational. The systemic symptoms include headache, lethargy, fatigue, sleepiness/sleeplessness, tender breasts, heavy lower abdomen, backache, painful knees, muscles, joints, inner thighs and swollen legs. The gastrointestinal symptoms includes increase or decrease of appetite, nausea, vomiting, and bloating. While the eliminational symptoms compromise constipation, diarrhea, frequent urination and sweating. On the other hand, the psychological symptoms include mood disturbances such as anxiety, depression, irritability, and nervousness [11, 16, 17].

Although the pathophysiology of dysmenorrhea has not been fully elucidated, current evidence suggest that the pathogenesis of dysmenorrhea is due to the increased secretion of prostaglandin F2α (PGF2α) and prostaglandin E2 (PGE2) in the endometrium, which are involved in increasing myometrial contractions leading to uterine ischemia and sensitization of pain fibers [1, 4]. The diagnosis of PD is made mainly by retrieving medical history and physical examination to ensure absence of pelvic pathology [1]. Menstrual cramps and intensity of pain are correlated to the high concentration of PGF2α and PGE2 in the endometrium, therefore the non-steroidal anti-inflammatory drugs (NSAIDs) are considered the cornerstone in the management of dysmenorrhea since they inhibit the action of cyclooxygenase (COX) [1, 18], an enzyme responsible for the production of prostaglandins.

Marjoribanks et al. conducted a review including 80 randomized controlled trials (5820 women), and they concluded that NSAIDs are 4.5 times more effective than placebo for pain relief (odds ratio [OR] = 4.37, 95% confidence interval [CI] = 3.76–5.09), two times more effective than paracetamol (OR = 1.89, 95% CI = 1.05–3.43), and there was no superiority of any individual NSAIDs for pain relief [18]. However, NSAIDs were associated with adverse effects (OR = 1.29, 95% CI = 1.11–1.51) including gastrointestinal (OR = 1.58, 95% CI = 1.12–2.23) and neurological adverse effects (OR = 1.58, 95% CI = 1.12–2.23) [18].

Hormonal contraceptives (HCs) are also considered first-line therapy for the management of dysmenorrhea unless contraindicated. The HCs are considered upon patient’s preference and desire for contraception, or for those who cannot tolerate or are not responsive to NSAIDs [19, 20]. HCs containing estrogen and progesterone, are effective in managing PD since they supress ovulation and endometrial proliferation, and also block the production of the prostaglandins [1]. It was reported that combined oral contraceptives (COC) of estrogen-progestin has been the most common method utilized over other hormonal contraceptives by dysmenorrheic females. In a longitudinal study, COC was proven to significantly decrease the severity of PD [21, 22]. However, the rate of use of COCs among dysmenorrheic females is not yet established, although a study has shown that the majority of females take COCs for pregnancy prevention, and only 14% use them for non-contraceptive reasons, including acne, and primary and secondary dysmenorrhea [23].

Several non-pharmacological interventions were recommended to manage PD, which can be either employed solely as an alternative therapy or as a complementary therapy [19, 24]. The use of non-pharmacological interventions is common among dysmenorrheic females. A recent meta-analysis, comprising 12,526 dysmenorrheic females, has revealed that 51.8% of females adopted different non-pharmacological measures to cope with their menstrual pain [25]. Exercise and topical heat application were proven to significantly reduce menstrual pain, and their efficacy was comparable to that of NSAIDs [26, 27]. These interventions were hypothesized to reduce menstrual pain by several mechanisms including increasing pelvic blood supply, inhibiting uterine contractions, and stimulating the release of endorphins and serotonin [25, 27–29].

Although dysmenorrhea is a common gynecological complaint among young females and has a significant impact on the quality of life, the menstrual disorders in the Middle East have received scanty attention, and PD is usually neglected. Dysmenorrheic females are not provided with sufficient information and this condition is left inadequately addressed. To our knowledge, previous studies have not thoroughly investigated the management-seeking practices, and the perceived effectiveness of various treatments in relieving pain. Therefore, this study aimed to address these gaps in order to gain a broader understanding of the prevalence of PD, its risk factors, associated symptoms, impact on daily activities, the management practices, as well as their perceived effectiveness. The study outcomes would inform the design of future interventions aiming at educating dysmenorrheic females on the appropriate use of therapies.

## Methodology

### Study and sample design

This observational cross-sectional study was conducted in six selected Lebanese universities using a convenience sampling approach between April and July 2019. The selected universities have a diverse student body, from all over Lebanon, with different backgrounds, cultures, and financial statuses.

### Inclusion and exclusion criteria

The inclusion criteria consisted of under or post-graduate university female students, aged between 18 and 30 years and having menses during the past six months. Excluded subjects were females having any of these conditions: (1) pathological pelvic conditions including endometriosis, chronic pelvic inflammatory disease, adenomyosis, polycystic ovarian syndrome, endometrial fibroids/polyps, or sexually transmitted diseases; (2) history of pelvic or abdominal surgery; and (3) receiving anti-depressants or anxiolytics since they modulate pain sensation [30].

### Questionnaire development and structure

A questionnaire was developed to collect information after an extensive literature review of relevant studies having similar aim and objectives [31–35]. This questionnaire consisted of 55 items, varying between close-ended questions (with pre-specified options) and open-ended ones. The questionnaire has four main sections, where the first three sections were filled by both dysmenorrheic and non-dysmenorrheic females, and the last section was completed by dysmenorrheic females only.

The first section gathered sociodemographic information, lifestyle habits and medical history. The second section retrieved information about the menstrual pattern, including the age at menarche, regularity and length of cycle, duration of menstrual bleeding, and heaviness of bleeding. The third section identified the characteristics of dysmenorrhea including its onset, duration of pain, severity, associated symptoms, and its impact on daily activities and academic performance. The severity of dysmenorrhea was measured using a 10-point visual analogue scale (VAS), a previously well validated and reliable scale [31, 32, 36, 37]. The VAS represents the female's perception of the degree of pain, ranging from having no pain to unbearable pain (0–10, respectively). The scales’ scores were classified as follows: 1–3 was considered mild pain, 4–7 moderate pain, and 8–10 severe pain [36–38].

The fourth section obtained the management-seeking practices, including consulting healthcare providers, the adopted non-pharmacological measures, and medications received to manage PD. Moreover, the self-perceived effectiveness of the practiced strategies was obtained using a 10-point VAS.

Two experts in the field reviewed the questionnaire for face and content validity. The experts assessed the questionnaire items relevance, specificity, and comprehensiveness. A pilot test was then conducted on 20 university students to assess the clarity, understandability, and organization of the constructed questionnaire. The participants were requested to evaluate its structure, length, and give their overall impression. The time for answering the whole questionnaire by each participant was recorded and amendments were made accordingly. After two weeks, the questionnaire was retested on the same sample to ensure its reliability and reproducibility. The data obtained from the pilot test was not included in the final data analysis.

### Sample size calculation

The sample size was calculated using the following formula for cross-sectional studies developed by Daniel et al. [39]. Where *n* is the sample size, *Z* is the Z value for 95% confidence limits (which is 1.96 when *α* = 0.05), *P* is the estimated prevalence of dysmenorrhea (which is 80%), and *d* is the desired precision (which is 4% in this study). The required sample size was 400 and increased by 20% for possible dropout and incomplete responses. Thus, the estimated sample size was 480.

$$n = \frac{{Z}^{2}P\left(1-P\right)}{{d}^{2}}$$

### Data collection

Two researchers visited the selected universities and approached females either in ground halls or in university cafeterias and invited them to participate in the study. The researchers explained the nature and purpose of the study and provided participants with cosmetic samples as incentives. Participants were reassured that their collected data will be kept confidential and anonymous. After obtaining their informed consent, they were asked to complete the self-administered questionnaire, and the approximate time spent in completion was 10 min. 

### Data preparation and analysis

After the data collection process, the filled questionnaires were screened for completeness, legibility, consistency, and clarity. Questionnaires filled with illegible, inconsistent, and incomplete answers were discarded. The dataset of valid questionnaires was coded and entered in an electronic spreadsheet. Then the data was reviewed, cross-checked with the original records, and cleaned from abnormalities, inconsistencies, outliers, and odd patterns.

Data were analyzed using the IBM Statistical Package for the Social Sciences (SPSS^®^) software version 24. The descriptive data were represented using frequencies and percentages for categorical variables and mean with standard deviation for continuous variables. Appropriate inferential statistical tests were used according to the type of research questions (comparison of means, testing association/relationship), type of variables (e.g., categorical, continuous), and distribution of data (normal or skewed). All continuous variables were tested for normality by the Shapiro–Wilk tests before statistical comparisons. Univariate analysis that tests the associations between dysmenorrhea status and categorical independent variables was assessed using Pearson Chi-square (χ^2^). Fisher’s Exact test was used to assess the association between medications used and adverse effects reported, and when the expected value within the cell is less than 5. Furthermore, the paired-sample t-test was conducted to evaluate whether the pain score decreased significantly after adopting non-pharmacological therapy and after receiving medications. Additionally, one-way analysis of variance (ANOVA) with Bonferroni post hoc test was used to compare the efficacy of different individual medication being utilized.

Bivariate logistic regression, using backward stepwise analysis, was utilized to assess the association between different significant independent variables with dysmenorrhea, and the adjusted odds ratio (AOR) was calculated. Moreover, multiple linear regression, using backward stepwise analysis, was computed to compare the means of the magnitude of analgesia using different non-pharmacological measures. Results with a *P* value < 0.05, with a 95% confidence interval, were considered significant.

### Operational definitions

#### Dysmenorrheic females

Participants who reported experiencing menstrual pain, with no pelvic pathology, at least once within the last 6 months.

#### Family history of dysmenorrhea

Positive family history of PD, where a first-degree relative (either mother or sister), had a history of menstrual pain [40].

#### Academic specialization

Participants enrolled in different faculties and majors were categorized as medical or non-medical students. Medical students (from faculties of pharmacy, medicine, nursing, dentistry, and applied health sciences) were supposed to have medical knowledge about common health conditions. Non-medical students were those who were studying in other faculties of non-medical disciplines.

#### Pain score

Indicates the self-perceived pain intensity of PD, which was measured using VAS.

- Pain score 1 = Dysmenorrhea pain intensity prior adopting any management strategy
- Pain score 2 = Dysmenorrhea pain intensity after adopting non-pharmacological measures
- Pain score 3 = Dysmenorrhea pain intensity after receiving a medication or an analgesic

#### The magnitude of analgesia

It estimates the treatment response of different adopted management strategies, including the non-pharmacological measures and the received medications. It is assessed by calculating the mean pain intensity difference of the self-perceived effectiveness prior and post-treatment, using the VAS. A positive difference is indicative of improvement from the utilized strategy.

- The Magnitude of analgesia of non-pharmacological measures:
  $$Pain\, Intensity\, Difference\, I\, \left(PID I\right) = Pain\, score \,1 -Pain\, score \,2$$
- The Magnitude of Analgesia of the medication used:
  $$Pain\, Intensity\, Difference\, II\, \left(PID II\right) =Pain\, score\, 1 -Pain\, score\, 3$$

### Ethical consideration

The World Medical Association Declaration of Helsinki guidance was followed in designing and conducting this study. The study was approved by the Beirut Arab University (BAU) institutional review board (IRB). Students were asked to participate voluntarily, and a written informed consent was obtained from them before administering the questionnaire (see the Additional file 1).

## Results

### Distribution of the participants

A total of 572 female students were approached in this study and 550 completed and returned the survey, making a response rate of 96%. The participants were recruited from six different universities as follows: Beirut Arab University (n = 182, 33.1%), Lebanese University (n = 80, 14.5%), American University of Beirut (n = 77, 14%), Lebanese American University (n = 74, 13.5%), Lebanese International University (n = 69, 12.5%) and Saint Joseph University (n = 68, 12.4%). Half of the participants were medical students (n = 275, 50%) and the remaining were non-medical students (n = 275, 50%).

### Socio-demographic data and lifestyle habits of the participants

As seen in Table 1, the mean age was 21.8 ± 2.7 years, and the majority of the participants were younger than 25 years (84.2%). Most of them were single (n = 514, 93.5%), and had not yet given birth to a child (n = 522, 94.9%). Almost half of the participants (n = 288, 52.4%) had a family history of dysmenorrhea (mothers or sisters). Almost half of the participants reported having a sedentary lifestyle (n = 295, 53.9%), and only 16 (2.9%) exercise on daily basis. Moreover, half of the participants had a history of weight loss attempts within the past year (n = 299, 54.4%).

**Table 1:** Socio-demographic characteristics, lifestyle habits and the menstrual pattern of the study participants (N = 550)

Characteristic	n (%)^a^	Dysmenorrhea		*P*^c^
		No n = 105 (19.1%)^b^	Yes n = 445 (80.9%)^b^	
Academic specialization				0.003^d^
Medical	275 (50)	39 (14.2)	236 (85.8)	
Non-medical	275 (50)	66 (24)	209 (76)	
Academic level				0.46
Undergraduate	478 (86.9)	89 (18.6)	389 (81.4)	
Postgraduate	72 (13.1)	16 (22.3)	56 (77.7)	
Age (mean = 21.82, SD = ± 2.77, range = 18–30)				0.11
18–24	463 (84.2)	83 (17.9)	380 (82.1)	
> 25	87 (15.8)	22 (25.2)	65 (74.8)	
BMI category (Kg/m^2^, mean = 22.2, SD = ± 3.38, range = 15.2–37.5)				0.21
Underweight (< 18.5)	59 (10.7)	7 (11.8)	52 (88.2)	
Normal weight (18.5–24.9)	394 (71.6)	84 (21.3)	310 (78.7)	
Overweight (25–29.9)	78 (14.2)	11 (14.1)	67 (85.9)	
Obese (≥ 30)	19 (3.5)	3 (15.8)	16 (84.2)	
Marital status				0.01
Single	514 (93.5)	92 (17.9)	422 (82.1)	
Married	36 (6.5)	13 (36.1)	23 (63.9)	
Parity				< 0.001^d^
No	522 (94.9)	93 (17.8)	429 (82.2)	
Yes	28 (5.1)	12 (42.8)	16 (57.2)	
Smoke				0.21
No	413 (75.1)	84 (20.3)	329 (79.7)	
Yes	137 (24.9)	21 (15.3)	116 (84.7)	
Alcohol				0.84
No	502 (91.3)	95 (18.9)	407 (81.1)	
Yes	48 (8.7)	10 (20.8)	38 (79.2)	
Family history of dysmenorrhea				< 0.001^d^
No	262 (47.6)	73 (27.8)	189 (72.2)	
Yes	288 (52.4)	32 (11.2)	256 (88.8)	
History of weight loss attempt within the last year				0.001^d^
No	251 (45.6)	63 (25.1)	188 (74.9)	
Yes	299 (54.4)	42 (14)	257 (86)	
Exercise (mean time = 1.32 h/week, SD = ± 2.55, range = 0–21)				0.01^d^
No	295 (53.6)	55 (18.6)	240 (81.4)	
Once to twice weekly	158 (28.7)	32 (20.2)	126 (79.8)	
Three to four times weekly	63 (11.5)	8 (12.7)	55 (87.3)	
Five to six times weekly	18 (3.3)	2 (11.2)	16 (88.8)	
On daily basis	16 (2.9)	8 (50)	8 (50)	
Menarcheal age (mean = 12.35 years, SD = ± 1.34)				0.32
9–11 (early onset)	129 (23.5)	20 (15.5)	109 (84.5)	
12–14 (Normative onset)	395 (71.8)	80 (20.2)	315(79.8)	
15–16 (Late onset)	26 (4.7)	5 (19.2)	21 (80.8)	
Period regularity				0.43
Irregular	77 (14)	12 (15.5)	65 (84.5)	
Regular	473 (86)	93 (19.6)	380 (80.4)	
Menstrual cycle length (mean = 28.52 days, SD = ± 3.51, range = 20–45)				0.09
21–35 days	520 (94.5)	103 (19.8)	417 (80.2)	
< 21 or > 35 days	30 (5.5)	2 (6.6)	28 (93.4)	
Length of menstrual flow (mean = 6.04 days, SD = ± 1.35, range = 2–14)				0.56
Normal (3–7 days)	504 (91.6)	98 (19.4)	406 (80.6)	
Abnormal (< 3 or > 7 days)	46 (8.4)	7 (15.2)	39 (80.8)	
The intensity of menstrual blood flow				< 0.001*
Light	31 (5.6)	13 (41.9)	18 (58.1)	
Moderate	412 (74.9)	86 (20.8)	326 (79.1)	
Heavy	107 (19.5)	6 (5.6)	101 (94.4)	

*BMI* body mass index, *SD* standard deviation

^a^Percentages for the columns

^b^Percentages for the row

^c^Univariate analysis was conducted to test the associations between variables with PD

^d^Statistically significant (*P* < 0.05)

### Menstrual pattern

Most of the participants had their onset of menarche at the age of 12 to 14 years with a regular period (n = 473, 86%) occurring on monthly basis. The mean length of the menstrual cycle was 28.5 ± 3.5 days, and the mean duration of menstrual bleeding was 6 ± 1.3 days. Most subjects had moderate menstrual blood flow (n = 412, 74.9%), whereas 107 (19.5%) had heavy blood flow.

### Prevalence of PD and its characteristics

The prevalence of PD among 550 university females was 445 (80.9%). Almost half of the females with dysmenorrhea suffered from frequent menstrual pain monthly (n = 246, 55.3%). Nearly two-thirds of the participants mentioned that their dysmenorrhea started within one year after menarche (n = 287, 64.5%). Almost half of the participants (n = 197, 44.2%) had menstrual pain 1–2 days before the period, and the other half (n = 248, 55.8%) reported that their menstrual pain occurs when the period begins. The mean duration of menstrual pain was 3.3 ± 2.3 days. The mean pain score among dysmenorrheic females was 6.47 ± 1.97. Most dysmenorrheic females described their menstrual pain as moderate 249 (56%) to severe 154 (34.6%).

### Physical and psychological symptoms associated with PD

The most commonly reported systemic symptoms by dysmenorrheic females were backache (n = 358, 80.4%), fatigue/lethargy (n = 274, 61.6%), muscle cramps (n = 274, 61.6%), tender breasts (n = 207, 46.5%) and headache (n = 183, 41.1%). As for the gastrointestinal symptoms, increase of appetite (n = 264, 59.3%), bloating (n = 179, 40.2%) and nausea/vomiting (n = 140, 31.5%) were commonly experienced. Moreover, diarrhea (n = 160, 36%), sweating (n = 98, 22%), and frequent urination (n = 90, 20%) were commonly reported as eliminational symptoms. Furthermore, the majority of females with PD (n = 421, 94.6%) reported experiencing mood changes during their painful periods. More than two-third of the participants (n = 348, 78.2%) mentioned that they experienced irritability and nervousness, while more than half of them (n = 253, 56.9%) experienced sadness, 162 (36.2%) felt anxious, and only 45 (10.1%) experienced social embarrassment.

### Risk factors of PD

The univariate analysis has found a significant association between physical activity and PD. Females who exercise on daily basis were less likely to suffer from PD (OR = 0.23, 95% CI = 0.08–0.64, *P* < 0.001). As shown in Table 2, binary logistic regression analysis has shown a significant association of PD with academic specialization, marital status, parity, history of weight loss attempt, blood flow, and family history of dysmenorrhea. Heavy blood flow was shown to be the most potent risk factor for PD (AOR = 10.28, 95% CI = 3.15–33.49, *P* < 0.001). Medical students were more likely to have PD than non-medical ones (AOR = 1.66, 95% CI = 1.01–2.72, *P* = 0.04), and females with a family history of dysmenorrhea had 2.5 times higher risk than those without (AOR = 2.52, 95% CI = 1.53–4.16, *P* < 0.001). Furthermore, females with a history of weight loss attempt had two times higher risk to have PD (AOR = 2.05, 95% CI = 1.26–3.34, *P* = 0.004). Finally, the major predictor that showed a significant difference in decreasing the risk of PD was parity by almost 90% (AOR = 0.18, 95% CI = 0.07–0.45, *P* < 0.001).

**Table 2:** Logistic regression analysis^a^ of significant risk factors associated with primary dysmenorrhea

Risk factor	B	SE	Wald	AOR	95% CI	*P*
Constant	− 2.4	0.55	18.78	0.08		< 0.001^b^
Academic specialization (reference: non-medical)						
Medical	0.50	0.25	4.10	1.66	1.01–2.72	0.04^b^
Parity (reference: nulliparous)						
Parous	− 1.68	0.45	13.79	0.18	0.07–0.45	< 0.001^b^
Family history of dysmenorrhea (reference: no)						
Yes	0.92	0.25	13.29	2.52	1.53–4.16	< 0.001^b^
Weight loss attempt (reference: no)						
Yes	0.72	0.24	8.47	2.05	1.26–3.34	0.004^b^
The intensity of blood flow (reference: light)						
Moderate (average)	1.06	0.43	5.96	2.89	1.23–6.79	0.01^b^
Heavy	2.33	0.60	14.95	10.28	3.15–33.49	< 0.001^b^

*AOR* adjusted odds ratio, *B* coefficient for the constant in the null model, *CI* confidence interval, *SE* standard error, *Wald* Wald chi-square test that tests the null hypothesis that the constant equals 0

^a^Binary logistic regression analysis was conducted using significant variables associated with dysmenorrhea, using backward stepwise analysis. Hosmer and Lemeshow test: 11.857

^b^Statistically significant (*P* < 0.05)

### Impact of PD on daily activities and productivity

Almost half of the participants with PD (n = 220, 49.5%) reported that menstrual pain had moderately to severely affected performing daily activities. Almost half of the dysmenorrheic females (n = 210, 47.2%) reported that PD has affected their studying ability, and the mean missed university days was 1 day (SD ± 1.34). Moderate/severe pain had a significant negative influence on daily activities (n = 213/403, 52.9%, X ^2^ = 19.92, *P* < 0.001), absenteeism (*P* < 0.001), and studying ability (*P* < 0.001) in relative to mild pain intensity.

### Management-seeking practices

#### Medical consultation

Out of 445 dysmenorrheic females, only one-third (n = 164, 36.9%) have consulted a healthcare provider. Pharmacists (n = 99, 60.4%) were most commonly consulted among the healthcare providers, followed by physicians (n = 57, 34.8%) and nurses (n = 8, 4.8%). There was no significant difference between medical (n = 97, 57.3%) and non-medical students (n = 70, 42.7%) in the consultation behavior (*P* = 0.09). Several reasons were reported by the females who did not seek formal medical advice. The most cited reasons were: dysmenorrhea is a normal physiological cycle (n = 199, 70.8%), painful periods can be tolerated (n = 105, 37.3%), and being a medical student with background knowledge about dysmenorrhea (n = 40, 24.9%). Other reported reasons included lack of time (n = 22, 7.8%), and that requesting a consultation about painful periods is embarrassing (n = 9, 3%). Moreover, females with dysmenorrhea had gained information about menstrual pain from their mothers (n = 198, 44.6%), friends (n = 38, 8.5%), sisters (n = 28, 6.2%), and internet resources/published media (n = 30, 6.8%).

#### Non-pharmacological measures adopted for managing PD

Three-fourth (n = 333, 74.8%) of the participants with PD adopted different non-pharmacological measures for pain management. The most common lifestyle measures being followed to relieve pain were sleeping (n = 207, 62.2%), resting (n = 159, 47.7%), increasing water intake (n = 157, 47.1%), drinking green tea (n = 93, 27.9), and applying heating pads (n = 90, 16.45%). The mean pain score after receiving non-pharmacological therapies (4.14 ± 1.985) was significantly lower than the mean pain score of PD before receiving non-pharmacological therapy (6.56 ± 1.956, *P* ˂ 0.001). As shown in Table 3, the multiple linear regression analysis has revealed that sleeping, applying heating pads, and intake of ginger and anise were significantly associated with a higher reduction in the pain score.

**Table 3:** Multiple linear regression analysis^a^ of the efficacy of non-pharmacological measures

Non-pharmacological measures	Unstandardized coefficients		Standardized coefficients	t	95% CI	*P*
	B	SE	β			
Sleeping	0.33	0.16	0.10	2.05	0.01–0.65	0.04^b^
Heating pads	0.66	0.17	0.19	3.71	0.31–1.01	< 0.001^b^
Ginger	0.74	0.35	0.11	2.08	0.04–1.43	0.03^b^
Anise	0.62	0.21	0.15	2.97	0.21–1.03	0.003^b^

*B* coefficient for the constant in the null model, *β* the standardized odds ratio, *CI* confidence interval, *SE* standard error, *t* the parameter estimate divided by its standard error

^a^Multiple linear regression analysis was performed using backward stepwise analysis, and the outcome was: Pain difference intensity I = (pain score 1) − (pain score 2). R: 0.372 and R^2^: 0.138, thus the model detects 13% of the variation in the mean of different pain score post-non-pharmacological measures. ANOVA F: 8.697, *P* value < 0.001

^b^Statistically significant (*P* < 0.05)

#### Medications used for managing PD

Most dysmenorrheic females reported taking medications to manage their pain 340 (76.4%). Of them 214 (62.9%) were self-medicated, whereas 126 (37.1%) were prescribed by healthcare providers. Pharmacists were the most common prescribers (n = 92, 73%), followed by physicians (n = 29, 23%) and nurses (n = 5, 4%). There was no statistically significant difference in taking medications for pain relief among medical (n = 189, 55.6%) and non-medical students (n = 151, 44.4%, OR = 1.54, 95% CI = 0.99–2.40, *P* = 0.15). Although most dysmenorrheic females reported taking medications to relieve PD (n = 340, 76.4%), 105 (23.6%) participants avoided the use of any pharmacological measure to manage their pain. Among those participants who do not take analgesics during PD, 51 (48.6%) showed a preference for lifestyle interventions, 43 (41%) did not believe that medications are necessary, 24 (22.9%) have a fear of medications’ side effects, and 24 (22.9%) can tolerate pain. Out of 340 females taking medications for pain relief, 90% reported taking analgesics, and only 10% reported taking antispasmodics. NSAIDs have accounted for 70.5% of the total medications being received, while paracetamol accounted for 11.5%, paracetamol/codeine/caffeine combination accounted for 5.5%, and a combination of paracetamol/NSAIDs accounted for 2.5%. The most common NSAIDs taken by dysmenorrheic females were mefenamic acid 500 mg (n = 89, 26.2%), followed by ibuprofen 400 mg (n = 85, 25%), ketoprofen 100 mg (n = 31, 9.1%), and diclofenac 100 mg (n = 23, 6.8%). Regarding the antispasmodics, butylscopolamine (hyoscine) and phloroglucinol/trimethylphloroglucinol have accounted for only 10% of the total medications being taken. In addition, 144 (42.4%) of dysmenorrheic females reported taking medications frequently, almost in every menses. Moreover, most participants reported taking analgesics either when the pain starts (n = 198, 58.2%) or when the period starts (n = 112, 32.9%). Most of the medications were administered orally (n = 305, 89.8%), followed by rectally (n = 20, 5.9) and parenterally (n = 15, 4.4%). These analgesics were administered once to twice daily, 134 (39.4%) and 158 (46.5), respectively, and the mean duration was 1.83 days, ranging from 1–7 days.

##### The magnitude of analgesia of medications

To assess the magnitude of analgesia of medications utilized, a paired-samples t-test was conducted**.** The results indicated that the mean pain score 3, after receiving medication, (2.16 ± 1.59) was significantly lower than the mean pain score 1, before receiving a medication (6.78 ± 1.88, *P* ˂ 0.001). In addition, the mean paired difference in the paired samples indicated that the pain score decreased on an average of 4.62 after receiving medications (95% CI = 4.42–4.82). A paired sample t-test was performed to analyze the efficacy of each medication in reducing pain, resulting in significant reduction with different medications used, regardless of dose, the pattern of use, and frequency of administration (*P* < 0.001). Ketoprofen was the most potent analgesic in which the mean pain intensity difference II was 5.74 ± 1.78, followed by diclofenac 5.56 ± 1.59, mefenamic acid 4.92 ± 1.92 and ibuprofen 4.51 ± 1.48. The ANOVA test revealed no significant difference among NSAIDs in their potency in reducing pain. However, post hoc comparisons using Bonferroni test showed that most NSAIDs, including mefenamic acid, ibuprofen, ketoprofen, diclofenac and naproxen were significantly more potent than paracetamol in treating PD (*P* < 0.001).

##### Adverse effects

Most of the participants denied experiencing any adverse effect (n = 285, 83.8%), whereas the rest of the participants (n = 55, 16.2%) reported experiencing adverse effects as sometimes (n = 39, 11.5%), most of the times (n = 13, 3.8%), and always (n = 3, 0.9%). The most commonly reported adverse effects from the utilized medications were abdominal pain (n = 36, 65.5%), drowsiness (n = 33, 60%), and flank pain (n = 9, 16.4%). Fisher’s Exact test was used to compare the association between the medications used and the adverse effects. The results revealed that all medications used, except mefenamic acid, had similar episodes of headache, diarrhea, constipation, drowsiness, and vomiting. Mefenamic acid, had a significant lowest risk to cause abdominal pain (n = 3, 8.3%, OR = 0.03, CI = 0–0.34, *P* = 0.005) when compared with ibuprofen (n = 9, 25%). However, mefenamic acid was associated with the highest risk of flank pain (n = 8, 88.9%, OR = 12, CI = 1.18–122.28, *P* = 0.02).

#### Comparison of magnitude of analgesia of non-pharmacological measures and medications used

The reduction in pain score after receiving medications was significantly higher than that after receiving non-pharmacological therapy. The mean pain intensity difference II (PID II, 4.73 ± 1.82) after receiving medications, was significantly higher than the mean pain intensity difference I (PID I, 2.42 ± 1.6), after receiving non-pharmacological measures (n = 262, mean difference = 2.3, 95% CI = 2–2.52, paired t-test = 21.45, *P* < 0.001).

## Discussion

The current study revealed a relatively high prevalence of primary dysmenorrhea among Lebanese university students. This finding is in line with several cross-sectional studies conducted in different countries including 89.6% in Lebanon [7], 84.2% in Lithuania [41], 88% in Australia [42], 85.1% in Palestine [43], 83.6% in Northern Ghana [44], and 84.1% in Italy [45]. This indicates that PD is a common public health issue that should be addressed appropriately. However, other studies have reported a lower prevalence rate of PD, such as 63.3% among nursing students in Southern Spain [46], 60% among 2721 women in Canada [47], and 70.6% in Saudi Arabia [48]. This variation in the prevalence rate among studies could be explained by several reasons including the absence of a universally accepted method to define PD, the selected age group of females, study populations themselves, perception of pain, cultural and social stress.

The majority of the participants described their PD as moderate to severe, which was in accordance with a study from Kuwait (82%), where most dysmenorrheic females had moderate/severe pain [43]. However, a Chinese study reported a lower percentage of females who described their pain as moderate to severe (65%) [49]. The variation in the intensity of pain among studies may be due to the difference in pain perception among participants in different countries and different scales being used to assess the pain severity.

The current study has also shown a negatively significant impact of dysmenorrhea on quality of life, where almost half of the dysmenorrheic females reported that their daily activities and studying ability were disrupted, and that they have missed at least one day at the university. This finding is consistent with a Jordanian cross-sectional study that revealed a significant association between severity of menstrual pain and the degree to which dysmenorrhea interfered with attending university (*P* < 0.001), and with engaging in social activities (*P* < 0.001) [50]. However, in an Ethiopian study, the impact of dysmenorrhea on quality of life and daily activities was much lower, where 14.8% of females reported limitation of activities, 16.2% missing classes, and 11% having poor concentration [51]. This variation might be due to cultural differences and pain tolerability among females in different countries.

Heavy blood flow was significantly associated with PD in the current study. This finding is consistent with a previous study [34]. Heavy menstrual blood flow might be considered a risk factor and a consequence of PD and this could be explained by the fact that PGs can disturb the homeostatic mechanism of the endometrium, hence increasing the blood flow [52]. Studies have also shown that women younger than 25 years are more likely to have PD, and the prevalence of dysmenorrhea falls with increasing age [41, 53]. However, although the prevalence in the current study was higher among females younger than 25 years, interestingly no statistical significance was detected among different age groups. This could be explained by the narrow age range among participants.

Moreover, medical specialization was revealed to be a major contributor to PD. Medical students were more likely to have PD than non-medical ones, which was consistent with a Palestinian study (OR = 2.2, *P* = 0.03) [43]. This could be interpreted by the academic pressure that medical students experience during their academic years, which in turn increases menstrual pain and decreases pain threshold. In addition, the current study has revealed that parity was significantly associated with a reduced risk of PD, which is consistent with the published finding of a systematic review [54]. The reduction or disappearance of menstrual pain after child delivery is hypothesized by the lower level of PGs released from the endometrium after term delivery and the neuronal degeneration in the uterus post-delivery [53]. Furthermore, smoking is considered a risk factor for PD. This is likely due to nicotine, which causes vasoconstriction, resulting in myometrial contraction and reduction in endometrial blood flow, which is common in dysmenorrhea [55]. An Australian cohort study, conducted on 9067 young females, who were followed between 2000 and 2012, found that smoking was significantly associated with menstrual pain (OR = 1.41), and more commonly attributed to initiation at an early age, below 15 years (OR = 1.50) [56]. However, in this study, no significant association was found between smoking and PD. This discrepancy can be explicated by the cultural differences since the incidence of smoking among adolescent females in Lebanon is uncommon, and the onset of smoking was not retrieved in the questionnaire, thus the participants who currently smoke might not be previously exposed to smoking for several years.

Positive family history of dysmenorrhea was also found to be a risk factor. This finding is consistent with several studies that determined positive family history as a significant risk factor for PD, with odds ratio varying between 3 and 20 [37, 53]. An explanation for this is that familial predisposition of PD could be due to genetic susceptibility among females with variant genotypes in several metabolic gene polymorphisms [57]. Another explanation for this may be that daughters of mothers with menstrual pain also may experience the same symptoms due to behavior learned from their mothers. Moreover, it could be due to a similar lifestyle and way of living. On the other hand, the current study also revealed that regular exercise reduced the risk of dysmenorrhea. Research had established that exercise decreases the level of stress, enhances pelvic blood flow and induces the release of endorphins, leading to a reduction in pain severity and duration of dysmenorrhea [58, 59]. Therefore, the importance of exercise and physical activity should be promoted among students, and physical education courses should be embedded in different programs in the universities.

Regarding the management-seeking practices, this study was consistent with the findings of previous studies in which females showed reluctance in seeking medical advice and preference for consulting family members, friends, and internet resources [13, 34, 44]. A recent qualitative study conducted among dysmenorrheic Spanish nursing students had investigated the reasons for not consulting healthcare professionals. The main reasons revealed were the normalization of menstrual pain, low expectation of the healthcare for menstrual pain, and preference for self-medication [3]. This could be explained by the fact that most females believe that period pain is a normal physiological condition that should be endured. This conception is enforced by family members, society and culture, resulting in females seeking self-care strategies rather than formal medical advice.

Although medical students represent better health knowledge and experience with chronic pain, there was no significant difference in their coping approaches with non-medical students. This finding was contradictory to the Hong Kong study, which revealed that medical students tended to use fewer medications than non-medical ones (75% vs. 91%, respectively. *P* = 0.002) [60]. This can be explained by pain perception differences among countries, and that medical students, as well as non-medical ones, tended to receive analgesics since they cannot tolerate pain and to be able to resume their normal daily activities.

Remarkably, none of the dysmenorrheic females, in the current study, received hormonal contraceptives to manage PD although they are recommended as first-line therapy [61]. This finding is consistent with the limited use of combined oral contraceptives (COCs) among unmarried females in the Middle East region [62, 63]. Commonly, the use of contraceptives among single females in the Middle East is looked at as taboo. In these conservative societies, with the prevailing Islam religion, sexual activity outside marriage is prohibited, and there is a common perception that COCs are used only for pregnancy prevention. Accordingly, reasons for avoiding the utilization of COCs among single females include sociocultural barriers, such as fear of being accused of sexual activity, fear of future infertility, and illiteracy. Therefore, future research is required to investigate females’ misconceptions about the use of contraceptive pills in the management of PD. Additional efforts should be made to raise awareness about the use of hormonal contraceptives and correct this misconception.

A recent Spanish study revealed that most dysmenorrheic females reported taking analgesics to cope with their menstrual pain, including NSAIDs, paracetamol, and anti-spasmodics [40]. Similarly, the majority of females in the current study, who reported taking medications for pain relief, took NSAIDs. Most of the dysmenorrheic females who reported taking analgesics for menstrual pain took mefenamic acid, ibuprofen, paracetamol, ketoprofen, and diclofenac. However, most of the females in a previous study reported taking mainly ibuprofen and diclofenac [51]. This existing difference in trend and pattern of analgesia use might be due to the supplies and availability of analgesics in different countries, drug prices, sources of analgesic recommendation, previous satisfying self-medication and home-left medications.

All medications used were effective in reducing the pain score, regardless of the appropriateness of their pattern of use, dose, and frequency of administration. Ketoprofen was the most potent analgesic in reducing the mean pain score, although no significant difference among different NSAIDs was detected. Post Hoc test revealed that most NSAIDs, including mefenamic acid, ibuprofen, ketoprofen and diclofenac were significantly more potent than paracetamol in treating PD (*P* = 0.001). This result was consistent with a systematic review of 80 randomized controlled trials (5820 women) that compared different NSAIDs with each other and revealed no superiority of any individual NSAIDs for pain relief. Moreover, NSAIDs appeared to be more effective in pain relief than paracetamol, while no difference in causing adverse effects [18]. Similar to the current results, the systematic review also reported that NSAIDs commonly cause adverse effects, including abdominal disturbance, headache, and drowsiness [18]. In the current study, mefenamic acid was the safest among different NSAIDS relative to causing abdominal pain. This result is consistent with a meta-analysis that concluded that mefenamic acid was associated with a low risk of side effects [64].

PD is attributable to an increase of prostaglandin synthesis and release in the uterus, about two days before the onset of menses, resulting in uterine contraction and pain. NSAIDs were recommended as initial therapy for managing PD by inhibiting the prostaglandin synthesis. NSAIDs are highly effective when initiated 1–2 days before the onset of period and continued through day two [18, 65]. However, in the present study most of the participants took analgesics when the pain started, which might contribute to lower efficacy and incomplete pain relief. Moreover, most of the females reported taking medications for pain relief once or twice daily, knowing that most of the medications being used for menstrual pain are recommended to be given every 6 to 8 h. These findings reveal that the females are receiving recommended medications for menstrual pain, but in suboptimal dose and the timing of NSAID administration in reference to menses is delayed. Accordingly, the inappropriate medication timing and dosage regimen would reduce the overall efficacy for pain relief [66]. Therefore, community pharmacists, being easily accessible healthcare professionals, have a unique role in educating females with PD and optimizing their treatment therapy. Pharmacists should guide dysmenorrheic females with the selection of appropriate over-the-counter (OTC) analgesics and advise them that they should be taken 1–2 days before the predicted menses onset rather than the as-needed basis to achieve satisfactory pain relief. Females should also be instructed to take NSAIDs every 6 to 8 h after meal intake, and not to exceed the recommended dose. Moreover, pharmacists should counsel these patients about the expected outcomes and side effects of the chosen analgesics. Furthermore, this study has established that the medications were superior to non-pharmacological measures in reducing the pain score. Thus, utilizing medications for pain relief is more effective in controlling dysmenorrheic pain and is considered a cornerstone in managing dysmenorrhea, while non-pharmacological measures can be adopted as adjunctive therapy and for patients who cannot tolerate analgesics.

This study has provided a panoramic image of PD since it has comprehensively covered different aspects of PD. It is a multi-center study that consisted of a representative sample, where findings may be generalizable to the young Lebanese female population. To the best of our knowledge, it is the first study performed in the Middle East that addressed the practiced management-strategies and their self-perceived effectiveness. However, the cross-sectional nature of this study is a potential weakness due to the possible introduction of recall bias, some covariates were not retrieved, and the content validity index of the developed questionnaire was not calculated. In addition, the study cannot prove the causality of predictors for PD but it, however, helped in generating a causal hypothesis. Thus, conducting a longitudinal study with a more standardized measurement of risk factors is needed for future research to estimate the actual effect and generate robust evidence.

## Conclusion

Primary dysmenorrhea (PD) is a common, overlooked gynecologic disorder characterized by menstrual pain associated with several physical and psychological symptoms that interfere with daily activities. It is typically treated with nonsteroidal anti-inflammatory drugs (NSAIDs) and hormonal contraceptives in addition to some non-pharmacological remedies. This study revealed that 81% of the Lebanese participants suffered from PD and that 35% of them reported severe pain. Exercising was found to reduce the severity of PD, whereas parity, family history of PD, weight loss, medical specialties, and severe menses were significantly associated with PD. Despite its adverse impact on females’ daily activities, academic performance, and psychological wellbeing, the majority of females did not seek formal medical advice, and they perceived it as a normal physiological cycle. Although most dysmenorrheic females received the recommended medications to manage their menstrual pain, however, the onset of medication initiation was delayed with respect to the menstrual period, and a sub-therapeutic dose was taken, resulting in suboptimal effectiveness for pain relief. Moreover, none of the participants declared the use of contraceptives for treating PD. This is probably due to the common perception of the Arab public that these medications are used only for contraception. Our findings reflect the lack of knowledge about dysmenorrhea and its optimal management. Therefore, educational programs and interventions tackling PD, its modifiable risk factors, and self-management support should be incorporated in universities to prevent unnecessary suffering and interruptions in daily activities. Moreover, there is an essential need for primary healthcare practitioners to provide counseling services about PD and to optimize its treatment outcomes.

## Supplementary Information

**Additional file 1.** Informed consent and questionnaire form.

## Data Availability

The dataset presented in this article is available only upon reasonable request, since it contains confidential information. Requests to access the datasets should be directed to the corresponding author (r.itani@bau.edu.lb).
