# Supplementary material for: Prevalence, risk factors, and management practices of primary dysmenorrhea among young females
Source: BMC Womens Health. 2021 Nov 8;21:392. doi: 10.1186/s12905-021-01532-w (PMC8576974; doi:10.1186/s12905-021-01532-w)
Supplement: Supplementary file 1 — Additional file 1. Informed consent and questionnaire form. [file 12905_2021_1532_MOESM1_ESM.pdf]

Additional File 1. Informed Consent and Questionnaire Form

Prevalence, Risk Factors, and Management Practices of Primary Dysmenorrhea among Young Females

Authors: Samar Karout, Lama Soubra, Deema Rahme, Lina Karout, Hani M J Khojah, and Rania Itani*

[1] Pharmacy Practice Department, Faculty of Pharmacy, Beirut Arab University, P.O.Box: 11-5020, Riad El Solh, 1107 2809, Beirut, Lebanon.

[2] Pharmacology and Therapeutics Department, Faculty of Pharmacy, Beirut Arab University, P.O.Box: 11-5020, Riad El Solh, 1107 2809, Beirut, Lebanon.

[3] Department of Radiology, American University of Beirut Medical Center, P.O.Box: 11-0236, Riad El-Solh, 1107 2020, Beirut, Lebanon.

[4] Department of Clinical and Hospital Pharmacy, College of Pharmacy, Taibah University, Madinah, Kingdom of Saudi Arabia.

***Corresponding author**: Rania Itani
r.itani@bau.edu.lb

Dear Respondent,

The following questionnaire is intended to investigate the prevalence of dysmenorrhea among Lebanese university students and assess the management practices. **Dysmenorrhea, painful periods**, is one of the most common **health disorders** among **females**. Dysmenorrhea is usually accompanied with **lower abdominal pain** and **discomfort** during **menstruation**. It negatively affects the patient's quality of life and may limit daily physical activities.

This questionnaire will take around 10 minutes to be completed. Your participation is important for us. Thank you for your time and cooperation in completing this survey.

Informed Consent

Your participation in this survey is **voluntary**, you have the right not to participate or withdraw at any time. This survey is intended for research purposes and your medical data will be kept confidential.

I voluntarily agree to participate in this research study. I have had the purpose and nature of the study explained to me in writing and I have had the opportunity to ask questions about the study. I understand that even if I agree to participate now, I can withdraw at any time or refuse to answer any question without any consequences of any kind and I understand that all the information I provide for this study will be treated confidentially.

Signature of the participant: --

Date: --

Questionnaire Form

Prevalence, Risk Factors, and Management Practices of Primary Dysmenorrhea among Young Females

Instructions: Please tick (√) for the answer and fill in the blanks when necessary

**Sociodemographic Information**
**1.** University: ________________
**2.** Faculty: ___________________
**3.** Specify your academic year:<br>□ 1st □ 2nd □ 3rd □ 4th □ 5th □ 6th □Post-graduate
**4.** Age: ______years
**5.** Weight: _____ kg
**6.** Height: _____ cm
**7.** Marital status: □ Single/Engaged □ Married □ Divorced
**8.** Do you have children: □ No □ Yes
**9.** Do you have any drug allergies? □ No □ Yes, specify: _____________
**Lifestyle Habits**
**10.** Do you smoke? □ No □ Yes □Ex-smoker<br>If **YES**: □ Cigarette □ Hookah (Nargila)<br>Number of Cigarettes per day: __ **OR** # of hookah session per day: ___
**11.** Do you drink alcohol? □ No □ Yes, occasionally □ Yes, on daily bases
**12.** Do you **exercise regularly**?<br>□ **No**, I don't exercise □Yes, **once** weekly □Yes, **twice** weekly<br>□Yes, **three** time weekly □Yes, **four** times weekly □Yes, **five** times weekly<br>□Yes, **six** times weekly □Yes, **every day**<br>If **YES**, specify the **type of exercise** you do: ____________________<br>specify the **time** you spend on exercising **per day**: ______minutes **OR** _____hours
**13.** During the **last year**, did you **try** to **lose weight**? □ No □ Yes
**Medical and Medication History**
**14.** Do you have any chronic medical disease(s)? □ No □ Yes, specify the condition:______________ ________
**15.** Do you have any gynecological (women-related) disease(s)? □ No □ Yes, specify the condition: ______________________
**16.** Do you have any gastrointestinal ulcers/bleeding? □ No □ Yes, specify the condition:________________
**17.** Do you take any prescribed medication(s) on daily basis? □ No □ Yes, specify: ____
**18.** Do you take any hormonal contraceptive pills? □ No □ Yes, specify:

	If **YES**, What is the reason behind using those contraceptive pills? ________________________________
**19.**	Do you take any over the counter (OTC) [Non-prescription] medication(s)? □ No □ Yes, specify: ____
**20.**	Do you take any herbal, supplements or gym products? □ No □ Yes, specify:
**21.**	Does your mother or sister(s) have history of painful periods? □ No □ Yes
	**Menstrual Pattern**
**22.**	At what age did you have your **first** menstrual cycle? ______years
**23.**	Do you have a **regular period** on **monthly basis**? □ No □Yes
**24.**	How many **days** are there between your **first day of one period** and the **first day of the next period** (**average menstrual cycle length**)? _______ days
**25.**	How long does your period **last** (duration of menstrual bleeding)? _____days
**26.**	How is the **blood flow** during your period (menses)?<br>□ Light (<1 sanitary pad soaked in 3 hours)<br>□ Moderate (>1 sanitary pad soaked in three hours)<br>□ Heavy (>1 fully soaked sanitary pad every two hours)
**27.**	Do you have **painful periods** characterized as **lower abdominal cramps** during menstruation?<br>□**No**, not at all □**Yes**, in some periods (**once** every **3 months**)<br>□Yes, in few periods (**once** every **6 months**) □Yes, in **all periods**
	**If your answer is <u>No, not at all</u> on the previous question, you have finished the survey.**<br>**Thanks for your time.**<br>**If your answer is YES, please continue the survey**

Dysmenorrhea Characteristics

28. When did you **start** experiencing painful periods?

□Within **3 months** of **puberty** □Within **6 months** of **puberty** □ Within **9 months** of **puberty**
□ Within 1 year of **puberty** □ Within **2 years** of **puberty** □ Within **3 years** of **puberty**
□ **>3 years** of **puberty**, specify ____________

29. Please **mark** the number that indicates the **severity** of your **period pain**. How would you **rate** your **menstrual pain** on a scale from **1 to 10**?

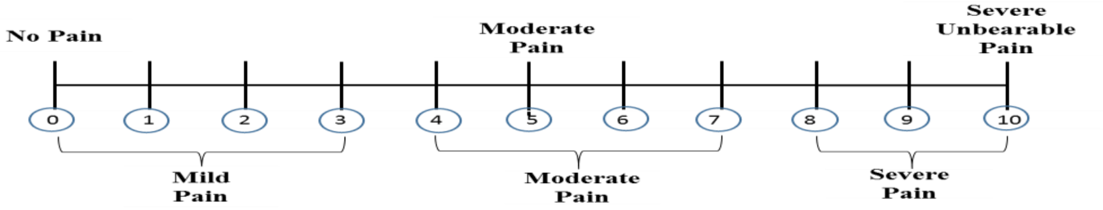

30. On which **day** of your cycle does the pain **<u>start</u>**?

□ **3 days before** your period begins □ **2 days before** your period begins
□ **1 day before** your period begins □ **1st day of your period**
□ **2nd day of your period** □ **3rd day of you period**

31. How **long** does the **pain last**? ______ hours **OR** _____days

32. Specify the **physical symptoms** associated with your painful period (**tick one or more options**):

Pain experienced:
□ Lower back pain □ Inner thighs pain □ Muscle cramps □ Painful breasts
□ Leg cramps

Systemic symptoms:
□ Nausea/ Vomiting □ Headache □ Fatigue □ Dizziness
□ Chills □ Sweating

Bowel Habit: □ Not affected □ Constipation □ Diarrhea

Urination: □ No change □ Frequent urination

Water retention:□ Non □ Bloating □ Weight gain
□ Swollen Legs

Appetite: □Not affected □ Loss of appetite □ Increase in appetite

□ If other(s), specify: _______________

33. Specify the **emotional symptoms** associated with your painful periods (**tick one or more options**):

□ **No change** in the emotional/mood state
□ Feeling **angry/irritable** in response to no specific reason
□ Feeling **restless/anxious** in response to no specific reason
□ Feeling **depressed/sad** in response to no specific reason
□ Feeling **socially embarrassed**

Impact of Dysmenorrhea on Quality of Life

34. How do your painful periods affect your **daily activity**?

□ **Unaffected** □ **Mildly** affected □ **Moderately** affected □ **Severely** affected

35. For the **last 6 months**, how many **university days** have you **missed** because of menstrual pain?

□**No**, never □Yes, **1** day □Yes, **2** days □Yes, **3-4** days □Yes, **5-6** days □Yes, **7 or more** days

36. How do your painful periods affect your **studying ability**?

□ **Unaffected** □ **Mildly** affected □ **Moderately** affected □ **Severely** affected

Management Seeking Practices

37. Who have you **consulted** about your painful periods?

Healthcare providers: □ Physician □ Pharmacist □ Nurse

Others:

□ Mother □ Sister □ Friend □ School/university instructor

□ Published media □ Internet resources

□ Other(s), specify: ________________________

38. If you **did NOT** consult a **heath care provider**, please specify the reason for **NOT to**:

□ Painful periods is a **normal physiological** cycle that occurs to females

□ Painful periods can be **tolerated**

□ Requesting a consult about painful periods is **embarrassing**

□ **Lack of time**

□ **Less availability** of female healthcare providers

□ Health care provider's consult is **not beneficial** in pain relief

□ Other(s),specify: ______________________

Lifestyle Measures for Pain Management

39. Do you follow any **lifestyle measures** to control your painful periods? □ No □ Yes

If **YES**, choose: (you can tick one or more of the following options)

Relaxing therapies:

□ Massage □Yoga □Exercise □Rest □Sleep □ Distraction by social media

□ Hot bath □Heating pad/Warm compress

Liquid intake: □ Increase **water** intake □ Drink **black** tea □ Drink **green** tea

□ Drink **chamomile** tea □ Drink **ginger** tea □ Drink **anise** Tea

□ Drink **cinnamon** Tea

□ Others, specify: ___________________________

40. Please **mark** the **number** that indicates the **severity** of your period pain **<u>after receiving</u>** the previously taken measure(s):

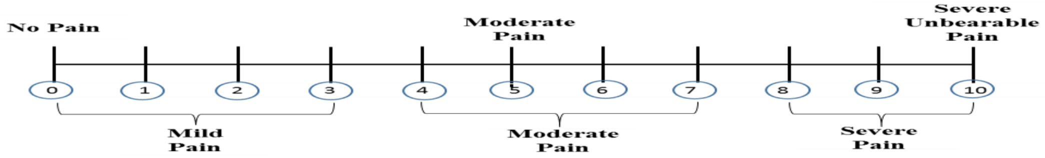

Medications Used

41. Do you take a **medication** to manage your **painful periods**?

□ No □ Yes, prescribed by a **physician** □Yes, recommended by a **nurse**

□Yes, prescribed by a **pharmacist** □ Yes, **without a prescription**

If your answer is <u>No</u>, Specify the reason(s) for not taking a medication to manage your painful periods **and you will be DONE with the survey, THANK YOU!** (you can choose one or more options):

□ Prefer **lifestyle** interventions

□ Don't believe that medications are **necessary**

□ Don't believe that medications can **help** in **relieving pain**

□ Fear of medication's **side effects**

□ Fear of **dependence** to medications
□ **Allergy** to medication
□ Presence of **medical reason** that **prevents** you from taking medication
□ Others, specify:__________________

42. If <u>**YES**</u>, What is the **name** of the **medication** used for painful periods:________________________

43. Please mark the number that indicates the severity of your period pain <u>**after receiving**</u> your <u>**medication**</u>**:**

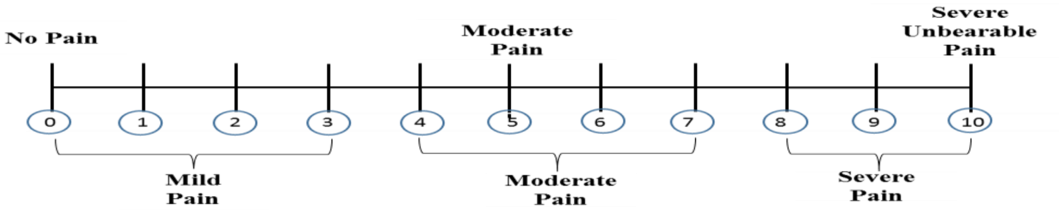

44. Within the **past 6 months**, how **often** did you take the **medication** for the management of your **painful periods?** □ In 1-2 periods □ In 3-4 periods □ In 5-6 periods

45. When do you **start taking medication** for the management of your painful periods?
□ **Two days before** your period starts □ **One day before** your period starts
□ When your **period starts** □ When you start **experiencing pain**

46. How **long** do you continue taking this medication? __________days

47. Specify the **route of administration:** □ Oral tablets or capsules □ Suppositories □ Injections

48. How **many times** do you take the previously mentioned medication **per day**?
□ **Once**/day (every 24 hours) □ **Twice**/day (every 12 hours) □**Three** times/day (every 8 hours) □**Four** times/day (every 6 hours) □**More**, specify: _______

49. Did you experience any **undesirable effect(s)** from the medication you used for the management of dysmenorrhea?
□No, never □Yes, sometimes □Yes, most of the time □Yes, always

50. If YES, what is (are) the **side effect(s)** you experience when taking the previously mentioned medication? (you can choose one or more options)
□ Stomach pain □ Headache □Diarrhea □ Constipation
□ Nausea/vomiting □Rash/allergic reactions □ Others, specify: __________________

Thank You!
